# The PCNA inhibitor AOH1996 impairs tumor growth and invasiveness in non–small cell lung cancer

**DOI:** 10.3389/fphar.2026.1802179

**Published:** 2026-03-30

**Authors:** Kholoud Arafat, Aya Mudhafar Al-Azawi, Shahrazad Sulaiman, Yomna Labanie, Samir Attoub

**Affiliations:** Department of Pharmacology & Therapeutics, College of Medicine & Health Sciences, United Arab Emirates University, Al-Ain, United Arab Emirates

**Keywords:** AOH1996, NSCLC, viability, colony growth, migration, invasion, tumor growth

## Abstract

**Background:**

Lung cancer (LC) is the leading cause of cancer-related deaths worldwide, with a 5-year survival rate of less than 10% in advanced or metastatic disease. AOH1996 is a novel small molecule inhibitor of cancer-associated Proliferating Cell Nuclear Antigen (PCNA). It disrupts DNA replication and repair by enhancing PCNA-RPB1 interactions, dissociating PCNA from transcribed chromatin, and inducing DNA double-strand breaks (DSBs), resulting in selective toxicity of various cancer types. The anticancer effects of AOH1996 on LC remain unclear.

**Methods:**

In this study, the anticancer effect of AOH1996 was first examined on four non-small cell lung cancer (NSCLC) cells, namely, A549, LNM35, NCI-H358, and NCI-H460 cell lines using cellular viability assay. Subsequent *in vitro* assays, including colony growth, migration and invasion assays in addition to tumor growth using chick embryo chorioallantoic membrane (CAM) assay *in vivo* were conducted on A549 and LNM35 cells. The underlying molecular mechanism has been investigated using western blots.

**Results:**

We demonstrated that AOH1996 significantly decreased NSCLC cells viability and colony growth *in vitro* in a concentration- and time-dependent manner. AOH1996 also suppressed their tumor growth in the chick embryo CAM xenograft model *in vivo* without causing toxicity. Moreover, AOH1996 reduced A549 and LNM35 cell migration and invasion *in vitro*. Mechanistically, AOH1996 induced cellular DNA damage, as evidenced by increased γH2AX levels. It also upregulated p53 and its downstream p21 along with activating caspase-3/7 and PARP cleavage.

**Conclusion:**

Our findings highlight AOH1996 as a promising therapeutic agent for NSCLC management, with potent effects on cell survival, migration, invasion, and tumour growth.

## 1 Introduction

LC is the most diagnosed cancer, with an estimated 2.5 million new cases, and a leading cause of cancer-related mortality, with an estimated 1.8 million deaths, worldwide in 2022 (Zhou et al., 2024). Histologically, NSCLC is the most prevalent subtype, accounting for 85%–90% of all LC cases (Li et al., 2023). Extensive research has been devoted to understanding the complicated and heterogeneous molecular pathology of this subtype (Smolarz et al., 2025). Consequently, multiple treatment modalities have been developed and approved, such as targeted and immunotherapy in addition to surgery, radiotherapy and chemotherapy (Smolarz et al., 2025; Jeon et al., 2025). Despite the wide scope of therapies, the prognosis remains poor with a low overall 5-year survival rate, specifically in advanced stages (Zhou et al., 2024). This alongside toxicities, resistance, and inqualities in accessing the current treatments highlight the need for deeper biological insights and the development of novel therapies (Li et al., 2023; Guo, 2022).

Proliferating cell nuclear antigen (PCNA) is a homo-trimeric ring protein located primarily in the nucleus of the proliferating cells (Søgaard and Otterlei, 2024). It encircles the DNA and plays a key role in cell cycle, DNA synthesis, and repair (González-Magaña and Blanco, 2020). Due to its integral role in cellular processes reflecting cell proliferation and DNA damage, PCNA is highly expressed in some tumors (Zeng, 2016; Lörz et al., 1994; Shin et al., 1993; Malkas et al., 2006), including NSCLC (Wang et al., 2018). Its high expression was found to be positively correlated with cancer stage, occurrence of lymphatic metastasis, and poor prognosis of NSCLC patients (Wang et al., 2018). Thus, PCNA is a potential target for NSCLC treatment.

(Gu et al., 2023) reported a novel small molecule inhibitor of cancer-associated PCNA, namely, AOH1996. It is an orally active and metabolically stable drug targeting transcription-replication conflicts (TRC) (Gu et al., 2023). AOH1996 selectively kills various types of cancers *in vitro* and *in vivo* by induction of cell cycle arrest and apoptosis (Gu et al., 2023; Wang Y. et al., 2025). The potency of AOH1996 was partially explained by its capability to stabilize the interaction of PCNA with RBP1 subunit of RNA polymerase II in addition to dissociating PCNA from actively transcribed chromatin. Subsequently, this causes RBP1 degradation, DNA replication collapse, and accumulation of double-strand breaks (DSBs) in cancer cells (Gu et al., 2023). Also, it was reported that AOH1996 upregulated the p-TBK1 and chemokines in head and neck squamous cell carcinoma (HNSCC), causing a reduction in the stemness and an increase in CD8^+^ T cell infiltration, respectively (Wang et al., 2025a). AOH1996 finds its way to the current recruiting phase I clinical trials for the treatment of refractory solid tumors (NCT05227326) and relapsed\refractory acute myeloid leukemia (AML) (NCT06763341). Taken the tumorigenic impact of PCNA in NSCLC, with the promising reported effects of AOH1996 in several cancer cell lines, the therapeutic effects of PCNA inhibition by AOH1996 on NSCLC remain unclear.

In this study, we aimed to investigate the therapeutic potential of AOH1996 in NSCLC cell, using adenocarcinoma A549 and large cell carcinoma LNM35 cell lines. Particularly, AOH1996 effect was evaluated on NSCLC cell viability, colony growth, migration, and invasion *in vitro* and tumor growth using the chick embryo CAM assay *in vivo* in addition to elucidating the underlying mechanism of action.

## 2 Materials and methods

### 2.1 Cell culture and reagents

Two human lung adenocarcinoma cell lines (A549 and NCI-H358) and two human large-cell lung carcinoma cell lines (NCI-H460 and its highly tumorigenic, invasive, and metastatic derivative LNM35 [NCI-H460-LNM35]) were used in this study. Cells were maintained in RPMI-1640 medium (Gibco, Paisley, United Kingdom) supplemented with 10% fetal bovine serum (FBS; Gibco, Paisley, United Kingdom) and Antibiotic-Antimycotic (10,000 units/mL of penicillin, 10,000 μg/mL of streptomycin, and 25 μg/mL of Gibco Amphotericin B) (Gibco, Grand Island, United States) at 37 °C in a humidified incubator with 5% CO_2_. The culture medium was replaced every 3 days, and cells were passaged once a week when reaching approximately 95% confluence. Cell viability was assessed by trypan blue dye exclusion and was maintained above 99% in all experiments. AOH1996 was purchased from MedKoo Biosiences (Morrisville, NC27560, United States). It was dissolved in DMSO and stocks were stored at −40 °C. At the day of each experiment, working solutions were freshly prepared in medium. Except the CAM assay, all experiments were performed in triplicate and repeated at least three times.

### 2.2 Cellular viability assay

Cells were seeded at a density of 5,000 cells per well into 96-well plates. After 24 h, the cells were treated for an additional 24 and 48 h with increasing concentrations of AOH1996 in triplicate. Control cultures were treated with the drug vehicle. The impact of AOH1996 on cell viability was determined using the CellTiter-Glo Luminescent Cell Viability Assay, which quantifies ATP as an indicator of metabolically active cells. The luminescent signal was measured using the GLOMAX Luminometer system. Data were presented as proportional viability (%) by comparing AOH1996-treated cells with the control cells, whose viability was defined as 100%.

### 2.3 Colony growth assay

Cells were seeded in six-well plates at a density of 50–100 cells per well. After 7 days of growth, pre-formed colonies were treated for an additional 10 days with AOH1996 at concentrations of 0.05, 0.25, and 0.5 μM. Colonies were then washed with PBS, fixed, and stained for 30 min with 0.5% crystal violet dissolved in methanol/distilled water (v/v). After staining, the colonies were washed three times with PBS, photographed, and counted. The percentage of colonies containing more than 50 cells was determined and expressed relative to the control-treated colonies, which were considered 100%. All experiments were performed in triplicate and repeated at least three times independently.

### 2.4 Cellular migration using the scratch-wound healing assay

Cells were cultured in 12-well tissue culture plates until reaching confluence. A linear wound was then made across the cell monolayer using a 200 μL sterile plastic pipette tip. The wells were then washed twice with PBS to remove detached cells and incubated at 37 °C in fresh medium containing 10% FBS, with or without AOH1996 treatment. Three arbitrary points at the top of each well were marked to ensure consistent measurement of the wound width. Images were captured at 0, 2, 6, and 24 h using an inverted microscope (Olympus 1X71, Japan; objective 4 ×). Migration was expressed as the mean ± SEM of the difference between the measurements at time zero and the 2, 6, and 24 h time periods considered.

### 2.5 Cellular invasion using Boyden chamber invasion assay

The invasiveness of control- and cells treated with AOH1996 was tested using Corning BioCoat™ Matrigel Invasion Chamber (8-µm pore size; Corning, NY, United States) according to the manufacturer’s protocol. Briefly, Cells (1 × 10^5^ cells in 0.5 mL 0.1% serum-containing medium) were seeded into the upper chambers of the system in the presence and absence of AOH1996 at the concentrations of 0.05, and 0.5 μM. The bottom chambers in the system were filled with media supplemented with 10% fetal bovine serum as a chemo-attractant and then incubated at 37 °C for 24 h. The non-penetrating cells were removed from the upper surface of the filter with a cotton swab. Cells that have passed through the Matrigel matrix and the 8-μm-pores insert were considered invasive and were quantified using the above-described viability assay. The assay was repeated three times for quantitative analysis.

### 2.6 Chick embryo CAM tumour growth assay

Fertilized White Leghorn eggs were incubated at 37.5 °C and 50% humidity. At the embryonic day 3 (E3), the CAM were dropped by drilling a small hole through the eggshell opposite to the air sac and aspirating 1.5–2 mL of the egg albumin. Then a 1-cm^2^ window were cut in the eggshell above the CAM. At day 9 (E9), cancer cells were trypsinized, washed with complete medium, and then suspended in equal amounts of Normal Saline (NS) and Matrigel® Matrix (Corning, Bedford, United Kingdom). A 100 μL inoculum of 1 × 10^6^ A549 cells or 0.1 × 10^6^ LNM35 cells were added onto the CAM of each egg, for a total of 14–16 eggs per cell line (to get sufficient surviving embryos at the end of the experiments). Two days later, tumours that began to be detectable were treated every second day at E11, E13, and E15 by dropping 100 μL of normal saline containing the AOH1996 or control solvent. At E17, the embryos were euthanized by topically applying 10–30 μL of Pentobarbitone Sodium (300 mg/mL, Jurox, Auckland, New Zealand). Next, the upper portion of the CAM were removed, transferred into PBS, and then the tumours were carefully cut away from normal CAM tissues and weighed to determine the impact of the treatments on tumour growth.

### 2.7 Western blot validation of targeted genes

Total cellular proteins from cells treated with AOH1996 were isolated using RIPA buffer (25 mM Tris. HCl pH 7.6, 1% nonidet P-40, 1% sodium deoxycholate, 0.1% SDS, 0.5% protease inhibitors cocktail (Sigma, Steinheim, Germany), 1% PMSF, and 1% phosphatase inhibitors cocktail (Thermo Scientific, Rockford, United States)). The whole-cell lysates were recovered by centrifugation at 14,000 rpm for 20 min at 4 °C to remove insoluble material, and 20 µg of proteins were separated by SDS gel for the validation of targets expression. After electrophoresis, the proteins were transferred on a nitrocellulose membrane, blocked with 5% non-fat milk and probed with primary antibodies pH2A.X (Millipore, United States, 1:1,000), Cleaved PARP (Cell Signaling, United States, 1:500), p53 (Santa Cruz, California, United States, 1:200), P21 (Cell Signaling, United States, 1:1,000), and β-actin-HRP (Santa Cruz, California, United States, 1:40,000) overnight at 4 °C. The blots were washed and exposed to secondary antibodies. Immunoreactive bands were detected using ECL chemiluminescent substrate (Thermo Fisher Scientific, Waltham, Massachusetts, United States), and chemiluminescence were detected using the LiCOR C-DiGit blot scanner (LI-COR Biotechnology, United States). Densitometry analyses were performed using an HP Deskjet F4180 Scanner with ImageJ software. The intensities of the bands were normalised to the intensities of the corresponding β-actin bands.

### 2.8 Statistics

Results were expressed as means ± S.E.M. of the number of experiments. The statistical analysis was performed using GraphPad Prism version 8.3.1 for Windows (GraphPad Software, San Diego, CA, United States). An unpaired t-test was used to assess the difference between two groups. One-way ANOVA followed by Dunnett’s multiple comparison test was used to compare 3 or more groups to the control group.

## 3 Results

### 3.1 Impact of AOH1996 on NSCLC cells viability and colony growth

**FIGURE 1:**
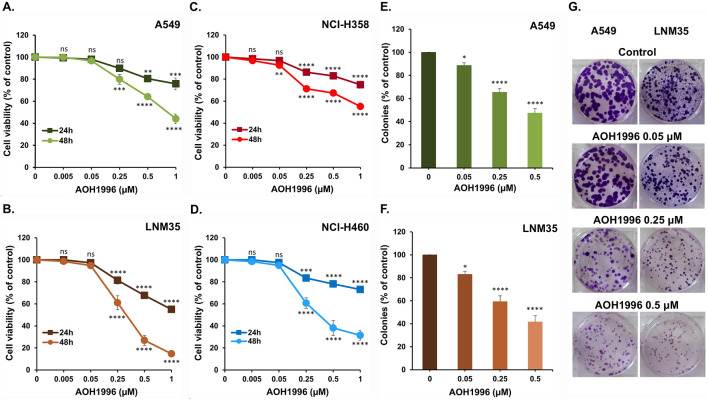
Impact of AOH1996 on NSCLC cells viability and colony growth. The viability of A549 **(A)**, LNM35 **(B)** NCI-H358 **(C)**, and NCI-H460 **(D)** cells treated with increasing concentrations of AOH1996 for 24 and 48 h. CellTiter-Glo® Luminescent Cell Viability assay was used to assess the cellular viability as described in the materials and methods. **(E–G)** A549 and LNM35 formed colonies were treated with increasing concentrations of AOH1996 every 48 h for 10 days. Colonies were fixed, stained, counted and photographed as described in the materials and methods. All experiments were repeated at least three times. Shapes and columns represent mean. Bars represent S.E.M. *Significantly different at P < 0.05, **Significantly different at P < 0.01, ***Significantly different at P < 0.001, ****Significantly different at P < 0.0001, ns (not significant).

To investigate the anticancer effect of AOH1996 on NSCLC, we utilized four NSCLC cells, namely, A549 and NCI-H358, representing the subtype of adenocarcinoma, in addition to NCI-H460 and LNM35, representing the subtype of large cell carcinoma. These cells were treated with increasing concentrations (0.005–1 µM) of AOH1996 for 24 and 48 h. As shown in Figures 1A–D, AOH1996 significantly reduced the cellular viability of respectively A549, LNM35, NCI-H3588, and NCI-H460. The inhibition is in a concentration and time-dependent manner with a remarkable decrease at 48 h. LNM35 and NCI-H460 cells showed more sensitivity to the AOH1996 concentration range compared to the A549 and NCI-H358 cells.

To further evaluate the anticancer potential of AOH1996 *in vitro*, its effect on the growth of A549 and LNM35 formed colonies was assessed using a range of concentrations (0.05–0.5 µM). Consistent with the viability data, AOH1996 significantly reduced the total number of colonies formed by A549 cells (Figures 1E,G) and LNM35 cells (Figures 1F,G) in a concentration-dependent manner. Taken together, these findings confirm the cytotoxic and growth-inhibitory effects of AOH1996 against NSCLC cells *in vitro*.

### 3.2 *In Vivo* antitumor activity of AOH1996 in the chick embryo CAM model

**FIGURE 2:**
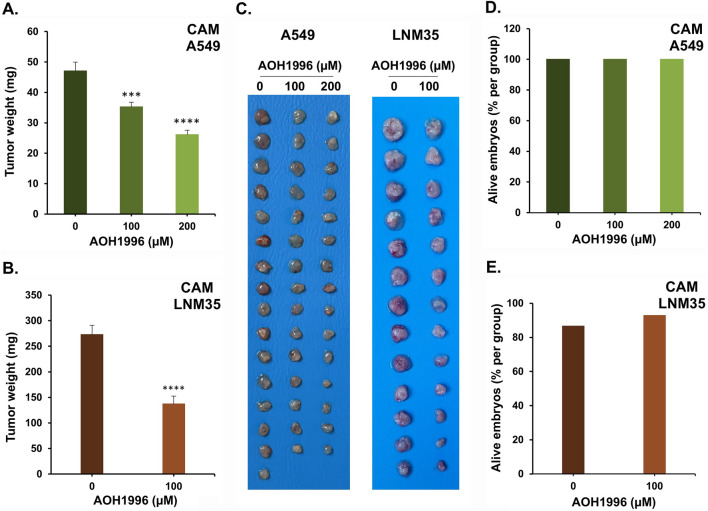
Effect of AOH1996 on NSCLC tumor growth using CAM Assay. **(A,B)** Tumor weights of A549 and LNM35 grafted on the CAM and treated with AOH1996 every 48 h for a total of 7 days. **(C)** images of the A549 and LNM35 tumors collected and photographed at E17. **(D,E)** Viability of the chick embryos in the A549 or LNM35 control and treated groups. Columns represent the mean of 14–16 eggs. Bars are S.E.M. ***Significantly different at P < 0.001, ****Significantly different at P < 0.0001.

To confirm the pharmacological relevance of our *in vitro* findings, we investigated the effect of AOH1996 on the growth of A549 and LNM35 tumors xenografted onto the chick embryo chorioallantoic membrane (CAM). Xenografted tumors were treated with AOH1996 at 100 µM or 200 µM every 48 h for 1 week. At the end of the treatment period, tumors were excised from the upper CAM and weighed. As shown in Figures 2A,C, AOH1996 reduced A549 tumor weight by approximately 26% at 100 μM and 45% at 200 µM. Notably, while 100 µM exerted a modest effect on A549 tumors, it reduced LNM35 tumor weight by approximately 50% (Figures 2B,C), indicating a higher sensitivity of LNM35 tumors to AOH1996. Importantly, the concentrations used did not induce apparent toxicity, as embryo survival rates were comparable between control and treated groups at the end of the experiment (Figures 2D,E).

### 3.3 Impact of AOH1996 on NSCLC migration *in vitro*

**FIGURE 3:**
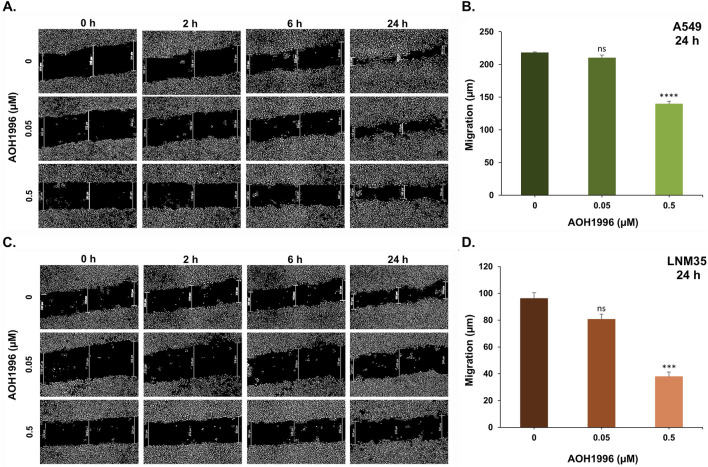
Impact of AOH1996 on NSCLC cells migration *in vitro*. Migration of A549 **(A,B)** and LNM35 **(C,D)** confluent monolayers cultured in the presence and absence of AOH1996 for 2, 6 or 24 h. The mean difference that cells migrated from the scratched area were measured using an inverted microscope (4x magnification). Experiments were repeated at least three times. Columns represent mean. Bars represent S.E.M. ***Significantly different at P < 0.001, ****Significantly different at P < 0.0001, ns (not significant).

Metastasis is a major contributor to poor prognosis, treatment failure, and cancer-related mortality (Fares et al., 2020). Approximately 40% of lung cancer patients present with metastatic disease at diagnosis (Morgensztern et al., 2010) and nearly 30% develop metastatic recurrence (Karacz et al., 2020). Therefore, targeting metastatic processes is of critical therapeutic importance. Since cell migration and invasion are key steps in the metastatic cascade (Fares et al., 2020), we investigated the effect of AOH1996 on NSCLC cell migration *in vitro* using wound-healing migration assay. As shown in Figures 3A–D, AOH1996 at a concentration of 0.05 µM slightly inhibited the migration of A549 (Panel A & B) and LNM35 cells (Panel C & D). However, treatment with 0.5 µM AOH1996 significantly reduced A549 migration with inhibition reaching 36% at 24 h. Similarly, it markedly decreased LNM35 cell migration, with inhibition reaching 60% at 24 h (Figures 3C,D).

### 3.4 AOH1996 suppresses NSCLC cell invasion independently of cytotoxicity

**FIGURE 4:**
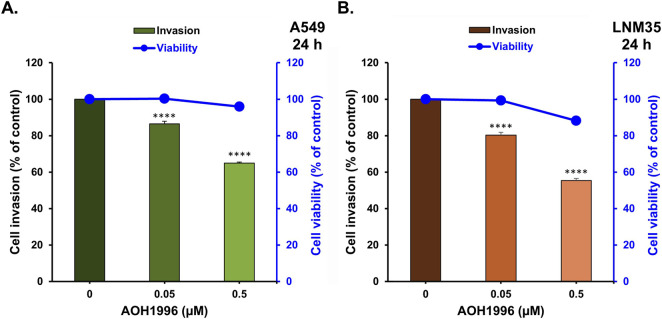
Effect of AOH1996 on the NSCLC cells invasion *in vitro*. Blue lines represent the viability of A549 **(A)** and LNM35 **(B)** seeded at a density of 100,000 cells/well and treated for 24 h with two concentrations of AOH1996 for 24 h. Columns represent the A549 **(A)** and LNM35 **(B)** cells that invaded the Matrigel and passed through the insert pores into the lower chamber. Cellular viability and invasion were determined using CellTiter-Glo® Luminescent Cell Viability assay as described in the materials and methods. All experiments were repeated at least three times. Shapes or columns with bars represent mean ± S.E.M. ****Significantly different at P < 0.0001.

The effect of AOH1996 on NSCLC cell invasion was next evaluated using a Matrigel Boyden chamber invasion assay. AOH1996 significantly reduced the invasion of both NSCLC cell lines in a concentration-dependent manner (Figures 4A,B). Treatment with 0.5 µM AOH1996 decreased invasion by approximately 35% in A549 cells and 45% in LNM35 cells. To ensure that the observed anti-invasive effect was not secondary to cytotoxicity, cell viability was assessed at cell densities comparable to those used in the invasion assay. As shown with the blue line, neither concentration of the AOH1996 concentrations tested affected cell viability, indicating that the inhibitory effect on invasion was independent of cell death.

### 3.5 Impact of AOH1996 on the DNA damage response (DDR) pathway

**FIGURE 5:**
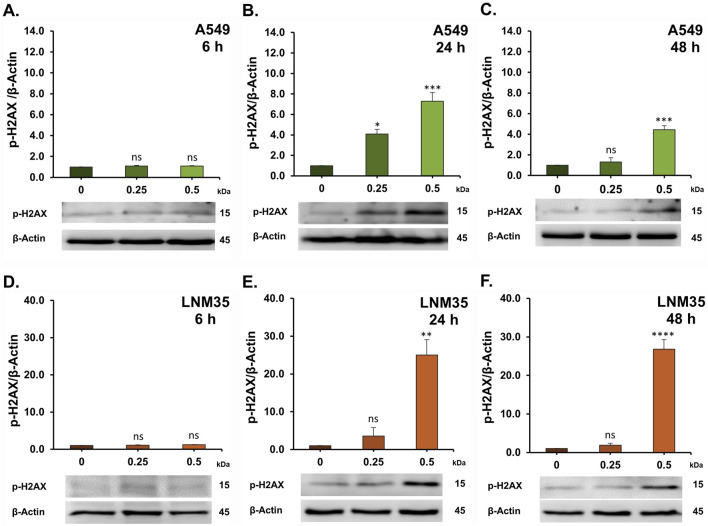
Western blot showing the effect of AOH1996 on the p-H2AX. **(A–F)** Quantification of Western blot showing the level of p-H2AX in A549 cells **(A–C)** and LNM35 cells **(D–F)** after treatment with AOH1996 for 6, 24, or 48 h. Experiments were repeated at least three times. Columns are mean. Bars are S.E.M. *Significantly different at P < 0.05, **Significantly different at P < 0.01, ***Significantly different at P < 0.001, ****Significantly different at P < 0.0001, ns (not significant).

**FIGURE 6:**
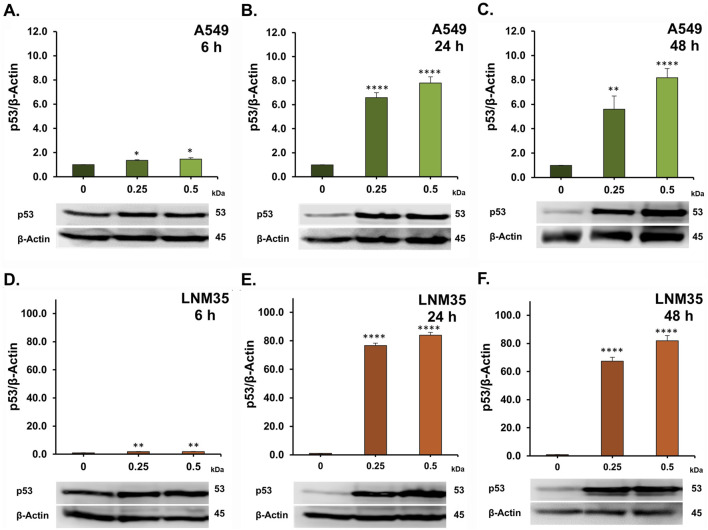
Western blot showing the effect of AOH1996 on p53. **(A–F)** Quantification of Western blot showing the level of p53 in A549 cells **(A–C)** and LNM35 cells **(D–F)** after treatment with AOH1996 for 6, 24, or 48 h. Experiments were repeated at least three times. Columns are mean. Bars are S.E.M. *Significantly different at P < 0.05, **Significantly different at P < 0.01, ****Significantly different at P < 0.0001.

**FIGURE 7:**
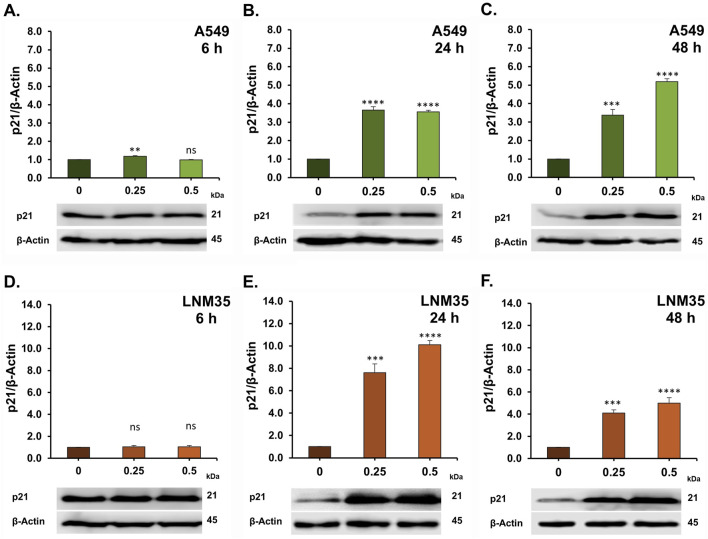
Western blot showing the effect of AOH1996 on the p21. **(A–F)** Quantification of Western blot showing the level of p21 in A549 cells **(A–C)** and LNM35 cells **(D–F)** after treatment with AOH1996 for 6, 24, or 48 h. Experiments were repeated at least three times. Columns are mean. Bars are S.E.M. **Significantly different at P < 0.01, ***Significantly different at P < 0.001, ****Significantly different at P < 0.0001, ns (not significant).

To explore the molecular mechanisms underlying the anticancer effects of AOH1996 in NSCLC cells, we investigated its impact on the DNA damage response (DDR) pathway. Phosphorylation of histone H2AX (γH2AX) is a well-established marker of DNA double-strand breaks (DSBs). Treatment with AOH1996 at concentrations of 0.25 and 0.5 µM markedly increased H2AX phosphorylation after 24 and 48 h in both A549 cells (Figures 5A–C) and LNM35 cells (Figures 5D–F). Consistent with the induction of DNA damage, these concentrations also significantly upregulated p53 expression (Figures 6A–F) and its downstream target p21 (Figures 7A–F) in both cell lines, indicating activation of cell cycle checkpoint and stress response pathways.

**FIGURE 8:**
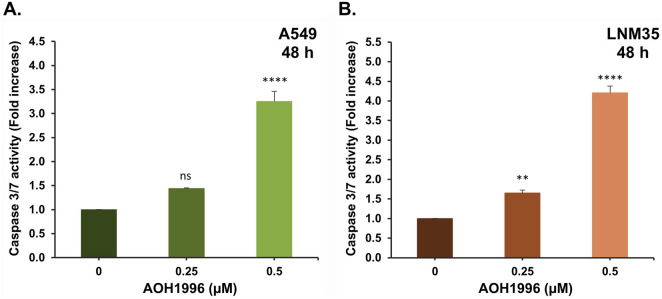
Impact of AOH1996 on caspase 3/7 activity in NSCLC cells. Caspase 3/7 activity in A549 **(A)** and LNM35 **(B)** cells treated with two concentrations of AOH1996 for 48 h. Caspase-Glo 3/7 Luminescent assay was used to assess the caspase 3/7 activity as described in the materials and methods. All experiments were repeated at least three times. Columns represent mean. Bars represent S.E.M. **Significantly different at P < 0.01, ****Significantly different at P < 0.0001, ns (not significant).

**FIGURE 9:**
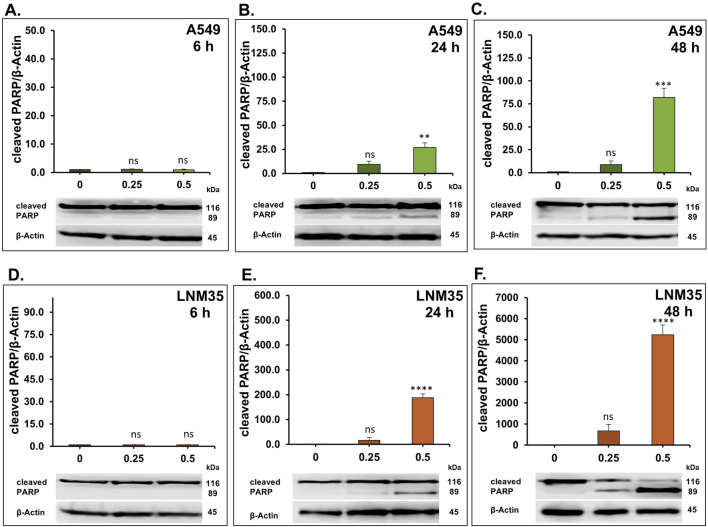
Western blot showing the effect of AOH1996 on the cleaved PARP. **(A–F)** Quantification of Western blot showing the level of cleaved PARP in A549 cells **(A–C)** and LNM35 cells **(D–F)** after treatment with AOH1996 for 6, 24, or 48 h. Experiments were repeated at least three times. Columns are mean. Bars are S.E.M. **Significantly different at P < 0.01, ***Significantly different at P < 0.001, ****Significantly different at P < 0.0001, ns (not significant).

To determine whether the observed DNA damage response and the inhibition of cell viability by AOH1996 were associated with the induction of apoptosis, caspase-3/7 activity, the key effector caspases, was assessed. A549 and LNM35 cells were treated with AOH1996 at concentrations of 0.25 µM or 0.5 µM for 48 h, after which caspase-3/7 activity was measured and normalized to the number of viable cells per well. As shown in Figure 8A, AOH1996 increased caspase-3/7 activity in a concentration-dependent manner in A549 cells. Treatment with 0.25 µM induced a non-significant 1.4-fold increase, whereas 0.5 µM resulted in a significant 3.3-fold increase. Comparable effects were observed in LNM35 cells, with a 4.2-fold increase in caspase-3/7 activity following treatment with 0.5 µM AOH1996 (Figure 8B). We further examined the effect of AOH1996 on PARP cleavage, a downstream target of caspase activation in the apoptotic pathway. Consistent with the observed increase in caspase-3/7 activity, AOH1996 markedly increased PARP cleavage at a concentration of 0.5 µM after 24 and 48 h of treatment in both A549 (Figures 9A–C) and LNM35 cells (Figures 9D–F).

Collectively, these findings demonstrate that AOH1996 induces DNA damage, activates the DNA damage response leading to cell cycle arrest and apoptosis in NSCLC cells.

## 4 Discussion

Advances in the understanding of LC biology, together with the successful integration of targeted therapies and immunotherapies into treatment protocols, have reshaped the therapeutic landscape of LC and improved clinical outcomes (Jeon et al., 2025). Nevertheless, LC remained the leading cause of cancer incidence and mortality worldwide in 2022, and its burden is projected to increase further over the next 2 decades (Zhou et al., 2024). This persistent burden, together with the limitations of current treatment strategies, underscore the need for novel therapeutic approaches (Li et al., 2023). PCNA represents a promising biomarker and therapeutic target in oncology because of its essential role in DNA replication, DNA repair, and regulation of chromatin structure (Wang et al., 2025b). A novel PCNA inhibitor, AOH1996, is currently undergoing phase I clinical evaluation for cancer therapy (Gu et al., 2023). AOH1996 is an orally bioavailable and metabolically stable small-molecule compound designed by (Gu et al., 2023) to selectively target cancer-associated PCNA (caPCNA) through binding to the PCNA-interacting protein box (PIP-box) (Gu et al., 2023). It demonstrated a promising anticancer effect across various types of cancer; however, its role in NSCLC remains underexplored. This study investigated the effects of AOH1996 on NSCLC cell viability, colony growth, migration, and invasion *in vitro*, as well as tumor growth in the chick embryo CAM model *in vivo*, while examining the underlying molecular mechanisms.

Our study demonstrated that AOH1996 (0.005–1 µM) induced a time- and concentration-dependent reduction in cell viability of the four NSCLC cell lines and inhibited the growth of pre-formed colonies in the NSCLC A549 and LNM35 cell lines. These findings align with the previously reported anticancer effect of AOH1996 in head and neck squamous cell carcinoma (HNSCC) (Wang et al., 2025a), acute myeloid leukemia (AML) (Kang et al., 2024), and pancreatic adenocarcinoma (PDAC) (Smith et al., 2025). In HNSCC models, AOH1996 (0.5–2.5 µM) reduced the proliferation of CAL27 and SCC15 cells in a concentration-dependent manner (Wang et al., 2025a). Similar concentrations also progressively decreased proliferation and methylcellulose colony formation in AML cell lines and in primary CD34^+^CD38^−^ blasts enriched for leukemia stem cells (LSCs) (Kang et al., 2024). In PDAC models, AOH1996 (0–20 µM) produced concentration-dependent cytotoxic effects across several KRAS-dependent cell lines (Smith et al., 2025). Notably, AOH1996 had greater efficacy in metastatic mouse cell lines derived from metastatic clones in KPCYX mice than in cell lines derived from non-metastatic clones, a difference attributed to higher MYC expression in metastatic cells (Smith et al., 2025). AOH1996 was initially screened for effects on cell viability in more than 70 cancer cell lines of diverse origins, including LC cells (Gu et al., 2023). Importantly, the concentrations used in the present study fall within ranges previously reported as non-toxic in normal cells. Particularly, AOH1996, at concentrations up to 10 μM, is non-toxic to human peripheral blood mononuclear cells (PBMCs), human small airway epithelial cells (hSAECs), and normal neural crest stem cells (Gu et al., 2023), and remains non-toxic up to 2 µM in normal CD34^+^CD38^−^mono nuclear cells enriched for hematopoietic stem cells (HSCs) (Kang et al., 2024).

To further validate our *in vitro* findings, we evaluated the antitumor effect of AOH1996 in an in vivo-like setting using the chick embryo CAM model of NSCLC. AOH1996, at doses of 100 and 200 μM, significantly suppressed tumor growth without detectable toxicity to chick embryos. Consistent with the *in vitro* results, LNM35 tumors were more sensitive to AOH1996 than A549 tumors. Notably, the antitumor effect of AOH1996 has not yet been investigated in NSCLC animal models, underscoring the novelty of our findings. Nevertheless, the antitumor activity and safety profile of AOH1996 have been demonstrated across multiple preclinical cancer models. Oral administration of AOH1996 at 50 mg/kg twice weekly for 3 weeks significantly reduced the growth of HNSCC (SCC15) subcutaneous tumors as well as orthotopic patient-derived xenografts (PDX) (Wang et al., 2025a). Similarly, twice-daily treatment with 40 mg/kg decreased tumor volume in xenograft models of neuroblastoma (SK-N-BE(2)c), breast cancer (MDA-MB-468), and small-cell lung cancer (H82) in ES1e/SCID mice (Gu et al., 2023). In PDAC models, administration of 100 mg/kg daily for 4 days reduced tumor growth in both orthotopic syngeneic and ectopic PDX models (Smith et al., 2025). Furthermore, the same dose given twice daily for 3 weeks reduced leukemia burden and prolonged survival in an AML PDX model (Kang et al., 2024).

Importantly, none of the above dosing regimens produced observable toxicity, as assessed by mortality, body weight loss, blood analyses, or other adverse effects. In GLP-controlled toxicity studies, the no-observed adverse effect level (NOAEL) exceeded 250 mg/kg/dose (twice daily) in mice and 75 mg/kg/dose (twice daily) in dogs (Gu et al., 2023). Furthermore, AOH1996 showed no mutagenic activity, as it did not induce frameshift or base-pair mutations in the Ames test (Gu et al., 2023). Collectively, these studies, together with our CAM data, support the broad antitumor potential of AOH1996 across multiple tumor types while reinforcing its favorable tolerability profile.

The differential sensitivity observed among the NSCLC cell lines used in this study may be partly explained by their distinct origins and biological characteristics. The A549 and NCI-H358 cell lines represent the lung adenocarcinoma subtype and were derived from a primary alveolar cell carcinoma and bronchioalveolar carcinoma, respectively (Lieber et al., 1976, Bairoch, 2018). On the other hand, LNM35 is a metastatic subline of the large-cell carcinoma NCI-H460 cell line, which was originally established from a pleural effusion of a patient with large-cell lung carcinoma (Bairoch, 2018, Kozaki et al., 2000). Cancer cells derived from metastatic sites may exhibit higher sensitivity to AOH1996. This possibility is supported by findings from (Smith et al., 2025), who reported enhanced sensitivity to AOH1996 in pancreatic cancer cells derived from metastatic lesions in KPCYX mice, an effect that was associated with elevated MYC expression (Smith et al., 2025). The contribution of MYC to the observed differential sensitivity in the present study remains unclear, as MYC expression in these cell lines has not been determined. The higher sensitivity of LNM35 cells to AOH1996 may be related to their highly proliferative and aggressive phenotype compared with A549 cells. Such characteristics may associate with increased PCNA expression, which could enhance responsiveness to the PCNA inhibitor AOH1996. This interpretation aligns with previous studies reporting a positive correlation between PCNA expression and tumor differentiation, size, metastasis, and stage in hepatocellular carcinoma Li et al. (2021) and NSCLC (Ye et al., 2020). Another potential explanation for the high sensitivity of LNM35 involves KRAS mutation status. Although both cell lines harbor KRAS mutations, they differ in the mutation type (Broad, 2025). Whether distinct KRAS mutations differentially influence the cellular response to AOH1996 in NSCLC remains unclear and warrants further investigation.

Metastasis is a fundamental hallmark of cancer, involving a complex multistep cascade that includes cells’ migration, invasion, intravasation, survival in the circulation, extravasation, and subsequent colonization in the distant sites (Fares et al., 2020). It is responsible for more than 90% of cancer-related mortality (Fares et al., 2020, Steeg, 2006). LC metastasis is associated with poor prognosis and limited clinical benefit from current therapies (Zhang et al., 2025). Therefore, the development of therapeutic strategies targeting this metastatic cascade remains a high priority. In the present study, AOH1996 at a concentration of 0.5 µM significantly inhibited migration and invasion of both A549 and LNM35 NSCLC cells *in vitro*. These findings align with those reported by (Wang et al., 2025a), who demonstrated that AOH1996 at 1 µM inhibited invasion of HNSCC CAL27 and SCC15 cells *in vitro* (Wang et al., 2025a). This anti-invasive effect was further validated *in vivo*, where AOH1996 treatment significantly reduced cervical lymph node metastasis of cancer stem cells isolated from an HNSCC patient-derived xenograft (PDX) model and orthotopically implanted into mouse tongues (Wang et al., 2025a). Collectively, these findings highlight the need for additional *in vivo* studies to specifically evaluate the impact of AOH1996 on NSCLC metastasis *in vivo*.

Mechanistically, Gu et al. (2023) reported that AOH1996 binds to the PCNA-interacting protein box (PIP-box) of PCNA, stabilizing PCNA’s interaction with the RBP1 subunit of RNA polymerase II (RNAPII) and promoting RBP1 degradation. AOH1996 was also found to dissociate PCNA from actively transcribed chromatin, leading to DSB accumulation (Gu et al., 2023; Smith et al., 2025). The anticancer effect of AOH1996 has also been attributed in part to cell-cycle arrest in the G2/M or S phases and induction of apoptosis without affecting normal cells (Gu et al., 2023; Smith et al., 2025). Consistent with these findings, our study showed that AOH1996 markedly increased H2AX phosphorylation in NSCLC cells, accompanied by upregulation of p53 and its downstream target p21. Phosphorylated H2AX (γH2AX) serves as a well-established marker of DNA DSBs (Prabhu et al., 2024). At the site of DNA damage, ATM phosphorylates H2AX, which will spread extensively around the break to form a unique H2AX foci that will regulate the DNA repair machinery (Podhorecka et al., 2010; Jurkovicova et al., 2022). ATM also regulates p53 either directly or through Chk2, leading to p53-dependent induction of p21, a CDK2 inhibitor that promotes cell-cycle arrest (Jurkovicova et al., 2022). Whether the effects of AOH1996 on γH2AX and p53 in NSCLC cells involve ATM signaling requires further investigation. In addition to its role in DNA damage signaling, H2AX also participates in apoptotic processes (Liu et al., 2008). In agreement with this role, our results showed that AOH1996 activates caspase 3/7, leading to PARP cleavage. Caspase 3/7 are executioner caspases activated during apoptosis to cleave essential proteins such as PARP (Julien and Wells, 2017). Cleaved PARP loses its DNA repair capacity, thereby facilitating the apoptotic cell death (Julien and Wells, 2017).

Collectively, our findings suggest that AOH1996 exerts its anticancer effects in NSCLC cells through DNA DSB-initiated signaling, resulting in cell cycle arrest and apoptosis. This comes in agreement with previous reports showing the phosphorylation of H2AX in AOH1996-treated KRAS (G12D)-expressing PDAC cells (Smith et al., 2025), HNSCC cells (Wang et al., 2025a), and neuroblastoma cells (Gu et al., 2023), leading to apoptosis and cell cycle arrest. In addition, DNA damage following AOH1996 treatment was previously confirmed in HNSCC (Wang et al., 2025a) and LC (HCC827) cells (Gu et al., 2023) using comet assay (Wang et al., 2025a).

In summary, this study demonstrates a promising anticancer effect of AOH1996 on NSCLC cell viability, colony growth, migration, and invasion *in vitro* in addition to tumor growth in chick embryo CAM. These effects appear partly mediated by the activation of DDR leading to the phosphorylation of H2AX, upregulation of p53 and its downstream p21 along with the activation of caspase 3/7 accompanied by PARP cleavage. Collectively, these findings provide a strong rationale for further preclinical investigations using a broader panel of NSCLC subtypes and animal models to better understand the differential sensitivity among NSCLC subtypes, elucidate additional mechanisms of action, and evaluate the impact of AOH1996 on metastasis *in vivo*. Moreover, exploring combination strategies of AOH1996 with current NSCLC treatments including chemotherapy, targeted therapies, and immunotherapy may enhance therapeutic outcomes.

## Data Availability

The original contributions presented in the study are included in the article/supplementary material, further inquiries can be directed to the corresponding author.
